# Predicting male dairy calf live weight for use in calf management decision support

**DOI:** 10.3168/jdsc.2021-0078

**Published:** 2021-07-01

**Authors:** F.L. Dunne, M.M. Kelleher, B. Horan, R.D. Evans, D.P. Berry

**Affiliations:** 1 Teagasc, Animal and Grassland Research and Innovation Centre, Moorepark, Fermoy P61 P302, Co. Cork, Ireland; 2 Irish Cattle Breeding Federation, Bandon P72 X050, Co. Cork, Ireland

## Abstract

The growing awareness and scrutiny of the management of young dairy calves, especially male calves, necessitates a support tool to aid in the planning of resource allocation on dairy farms. There is a desire among some vendors for a minimum calf weight when purchasing young dairy bull calves. Hence, the objective of the present study was to investigate whether live weight of young calves (approximately 10–50 d old) can be predicted using readily accessible animal-level features, especially features that may be available in advance of birth. A multiple linear regression mixed model was developed with the live weight of 602 dairy bull calves aged between 10 and 42 d as the dependent variable; the age at which an animal is predicted to reach a predefined live weight was then estimated based on the model regression coefficients. Fixed effects included in the multiple regression model were dam parity, gestation length, and parental average genetic merit for relevant traits available in Ireland; namely, birth weight, birth size, and carcass weight. Herd of origin was included as a random effect, with all calves having been sold directly from the farm of birth. Live weight data were recorded at the point of sale when calves were, on average, 26 d old with a mean live weight of 56.6 kg. Animals were randomly assigned to 10 separate (i.e., folds) cross-validation data sets without replacement (i.e., each fold consisted of a different 10% of the data to test the model, with the remaining 90% of data being used to train the model) to quantify the accuracy of prediction. Across all data, the correlation between actual and predicted live weight was 0.76; the regression coefficient of actual live weight on predicted live weight across all data was 0.99. The root mean squared error of prediction varied from 4.40 to 6.66 kg per fold. Across all data, the root mean squared error was 5.61 kg, implying that 68% of live weight predictions were within 5.61 kg of the actual live weight. Given the potential availability of all model features in advance of birth (gestation length can be predicted from ultrasound examination of the pregnant uterus, although substituting parental average genetic merit for gestation length had minimal effect on model performance), predictions can be integrated into a dairy farm decision support tool to aid in the management of labor and infrastructure resources to achieve minimum live weight specifications before sale.

## Highlights

• A robust yet simple statistical model for predicting young male dairy calf live weight was developed.
• The predictive model can be adapted to estimate the age when a calf reaches a target live weight.
• The model can be integrated into a decision support tool to inform calf labor and infrastructure management.

## Graphical Abstract

**Figure:**
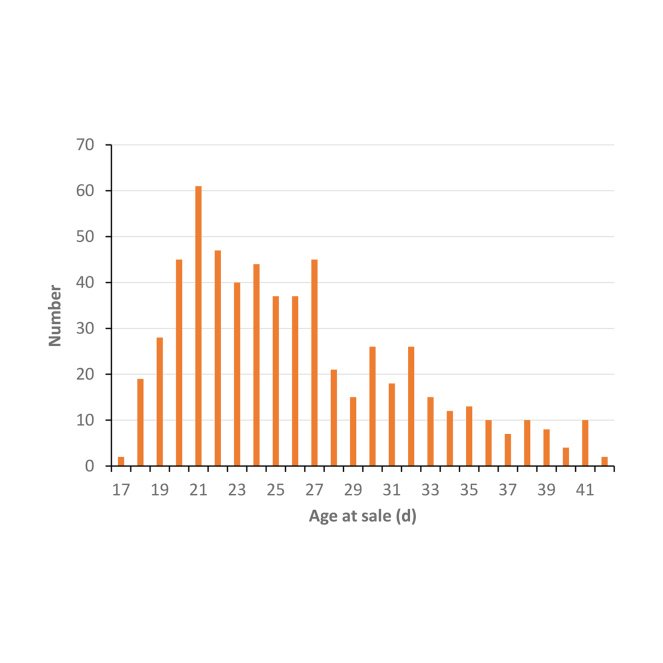
**Summary:** As the public scrutiny of dairy calf management and welfare intensifies, support tools to aid in farm workload and infrastructure management can be invaluable. Many purchasers of male dairy calves have minimum calf live weight thresholds. Being able to predict the age, and therefore date, that the male surplus dairy calves achieve this weight can be useful in planning; this is especially true if these predictions are available before the calf is born. Using estimates of genetic merit for a series of readily available traits, prediction of calf live weight was possible. A live weight predictive model was developed, trained, and tested using a 10-fold crossvalidation technique in order to quantify the accuracy and robustness of the predictions. The regression coefficients remained relatively consistent across each of the folds. With simple algebra, this prediction can be translated into the expected age when a calf will reach a target live weight.

Calf management on dairy farms can be resource intensive in terms of both skilled labor requirements and specialized infrastructural requirements. Dairy heifer calves are a valuable commodity (i.e., replacements for the dairy herd), but the supply of dairy bull calves generally far surpasses the demand; this therefore has implications for the value of dairy bull calves. A desire generally exists to rapidly sell bull calves, thus alleviating the pressure on resources during the calving season. Legislation governing the minimum age at which calves can be transported off farm has been imposed in some jurisdictions (Berry, 2021). Many purchasers of these bull calves actively seek minimum live weight thresholds. Achieving these live weight thresholds improves not only the marketability of the bull calves but also the price received. However, reaching heavier live weight generally requires a longer duration on farm with its associated costs and risks (e.g., mortality; Ring et al., 2018), including opportunity costs. Adequate planning is therefore paramount to ensure that ample housing and feeding facilities always exist to cater to all resident calves. This is particularly important for dairy herds in which a peak in calf births can occur, as in seasonal-calving production systems such as those in Ireland (Berry et al., 2013).

The objective of the present study was to construct a relatively simple statistical model to predict the live weight of dairy bull calves in the first 50 or so days of life. Of particular interest was to use features that could be available well before the calving season, including estimates of genetic merit of the calf for a series of traits related to animal size. The age at which a given live weight is expected to be reached can be predicted from the model solutions using simple algebra. Coupled with predictions of herd expected calving dates from service and pregnancy diagnosis data, it would then be possible to compute the expected number of dairy calves residing on the farm for any given day in the future.

Data were available on 602 male dairy calves aged 10 to 42 d. No formal sample size calculation was conducted because sample size was based on the available data. All calves were sold either through one of 6 Irish livestock auctions or directly from 3 research dairy farms between February and April 2020, and live weight was recorded at the time of sale. All calves were sold directly from the farm of birth. A total of 34 spring-calving herds from across Ireland were represented. Herd size was, on average, 135 milking dairy cows, varying from 34 to 506 dairy cows per herd; this was larger than the national average dairy herd size of 81 cows (Teagasc, 2019). Within the present study, the number of calves sold per herd varied from 4 to 60, with a median of 27 calves per herd. A total of 578 calves were Holstein-Friesian, and the remaining 24 calves were Holstein-Friesian × Jersey crossbreds. Confining the analysis to only the Holstein-Friesians had negligible effect on the conclusions; therefore, all animals were included. All calves had a known gestation length of between 266 and 291 d; the mean was 278.43 d. The EBV for birth size, birth weight, and carcass weight from the Irish national genetic evaluations were available for all calves.

Estimated breeding values for birth size and birth weight are generated in Ireland using a 6 × 6 multitrait, multibreed genetic evaluation that includes the direct (i.e., the animal's own genetic contribution to the phenotypic realization) and maternal (i.e., the dam's additional contribution to the offspring's performance) calving difficulty traits pertaining to dairy heifers, dairy cows, beef heifers, and beef cows separately as well as the direct and maternal birth size trait and the direct and maternal birth weight trait. Only direct EBV representing the animal's own genetic contribution for each of the respective traits were considered here. The parental average EBV for birth size and weight used in the present study were from the November 2020 national genetic evaluation, which included 1,932,005 phenotypic records for birth size and 199,759 phenotypic records for birth weight but did not include phenotypes of the calves used in the present study. The associated pedigree file contained 24,680,609 animals. Birth weight is measured using a weighing scale, whereas birth size is subjectively scored by producers on a scale of 1 (very small) to 5 (very large).

Estimated breeding values for carcass weight, conformation, and fat score in Ireland are generated using a 12 × 12 multitrait, multibreed genetic evaluation that includes a series of different live weight traits at various stages of life as well as cow live weight. Carcass weight in Ireland is measured, on average, 1 h after slaughter following the removal of the head, legs, thoracic and abdominal organs, and internal fats and hide. Carcass conformation and carcass fat are both recorded using the 15-point EUROP classification systems (Englishby et al., 2016) determined from video image analysis (Pabiou et al., 2011). Carcass conformation score reflects the shape and development of the carcass, particularly on the round, back, and shoulders. Carcass fat score represents the level of fat covering the carcass and within the thoracic cavity of the carcass. Scores of 1 for carcass conformation and fat score represent poor conformation and low fat cover, respectively, whereas scores of 15 represents the opposite (Englishby et al., 2016). Parental average EBV from the November 2020 national genetic evaluation that contained 9,572,024 carcass trait phenotypes were used, which did not include any phenotype of the animals from the present study. There were 23,837,155 animals in the associated pedigree file. The heritability for birth weight, birth size, and carcass weight used in the national genetic evaluations was 0.55, 0.71, and 0.60, respectively.

A linear regression model was developed in SAS (version 9.4; SAS Institute Inc.) manually using a forward and backward stepwise regression approach. The eventual model fitted was

[1] weight = age + parity + gestation + EBV_Bsize_ + EBV_Bwt_ + EBV_Cwt_ + herd + *e*,

where weight is the recorded live weight (kilograms) of the calf; age is the age (days) of the calf when weighed, included as a fixed effect; parity is the fixed effect of parity number of the calf's dam (levels included 1, 2, 3, 4, and 5+); gestation is the covariate of gestation length (days); EBV_Bsize_ is the covariate of birth size EBV; EBV_Bwt_ is the covariate of birth weight EBV; EBV_Cwt_ is the covariate of calf carcass weight EBV; herd is the random effect of the herd in which the calf was born; and *e* is the residual term. No nonlinear associations were detected. Neither EBV for carcass conformation nor EBV for fat cover were associated with calf live weight when included in the multiple regression model with the other model terms and so were not considered further, nor were EBV for live weight at later stages of life. Genetic and residual variance components for calf live weight were also estimated using a linear mixed model in Asreml (Gilmour et al., 2009). Fixed effects included in the model were herd of birth, dam parity, and calf age (days). A random term representing the direct additive genetic effect was included in the model. Relationships among animals were captured via the numerator relationship matrix, which included 27,127 animals related to the 602 calves.

The data set of 602 animals was randomly split into 10 smaller data sets using a random number generator, and a 10-fold cross-validation was undertaken to evaluate the model. Statistics used to determine the accuracy of prediction of live weight per fold included the following: (1) mean bias error in predicted live weight to test whether the model was over- or underpredicting the live weight based on a 2-tailed *t*-test comparing the mean bias error against zero, (2) root mean squared error (**RMSE**) to assess the variability of the residuals, (3) correlations to determine the strength of the linear relationship between the predicted and actual live weight and, (4) the linear regression coefficient of actual live weight on predicted live weight (the expectation was 1).

Mean calf live weight in the data set was 56.61 kg (SD = 8.66 kg) based on a mean calf age of 26 d (SD = 5.77 d). The model coefficients when applied to the full data set are shown in Table 1, and the variability in the regression coefficients across each of the 10 folds is shown in Figure 1. Of the data set used in the present study, 21, 22, 17, 13, and 28% were from dams in first, second, third, fourth, and fifth or greater parity, respectively. Relative to calves from primiparous dams, the relative mean calf live weight from dams in parity 2 to 5+ when adjusted for all other effects in the model was 2.74 (SE = 0.72), 2.66 (SE = 0.76), 4.6 (SE = 0.83), and 3.84 kg (SE = 0.69; Table 1), respectively. The heritability of calf live weight was 0.38 (SE = 0.14); therefore 38% of the phenotypic variation in calf live weight can be attributed to the additive genetic merit of the animal. Across all the data, live weight increased by, on average, 0.61 kg (SE = 0.05) per day of calf age. Live weight increased, on average, by 0.22 kg (SE = 0.06) per extra day of gestation length. The change in live weight per unit change in EBV for birth weight (kg), birth size (1-to-5 scale), and carcass weight (kg) when estimated from the multiple regression model was 0.75 (SE = 0.21), 0.18 (SE = 0.07), and 0.10 (SE = 0.03), respectively; when expressed in genetic standard deviation units, the respective values were 2.26, 0.09, and 1.89. The association between EBV and live weight strengthened when only one of the EBV was included in the model (Table 1).

**Table 1:** Model solutions (SE in parentheses) for the entire data set estimated using multiple regression (Estimate) or when each of the EBV for birth size (EBV_Bsize_), birth weight (EBV_Bwt_), and carcass weight (EBV_Cwt_) were individually included in the model along with age, dam parity, gestation length, and herd

Term	Estimate	EBV_Bsize_	EBV_Bwt_	EBV_Cwt_
Age	0.61 (0.05)			
Parity 1	2.74 (0.72)			
Parity 2	2.66 (0.76)			
Parity 3	4.60 (0.83)			
Parity 5+	3.84 (0.69)			
Gestation	0.22 (0.06)			
EBV_Bsize_	0.18 (0.07)	0.40 (0.06)		
EBV_Bwt_	0.75 (0.21)		1.28 (0.17)	
EBV_Cwt_	0.10 (0.03)			0.19 (0.03)

**Figure 1:**
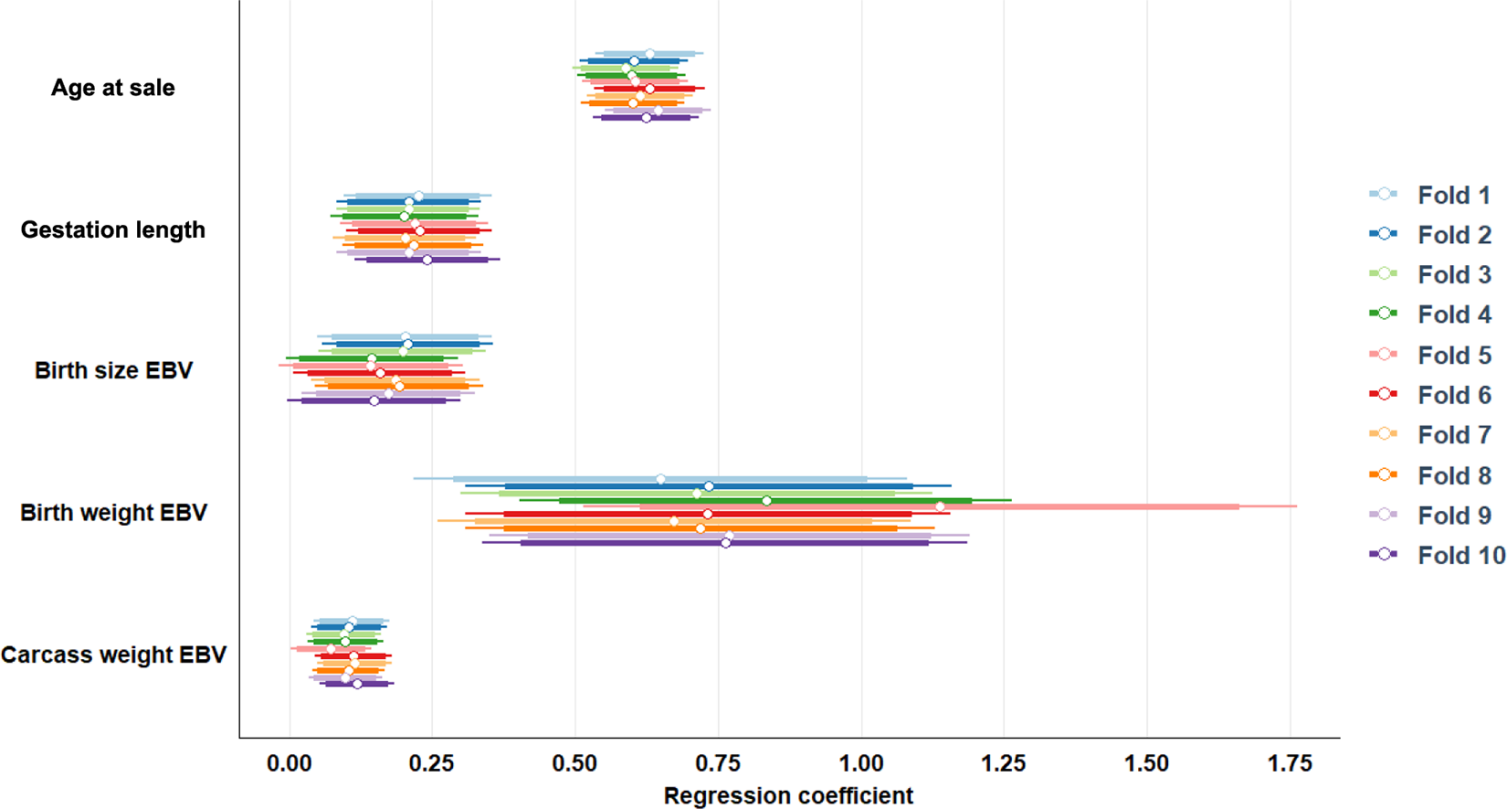
Forest plot of the multiple regression model solutions estimated for the covariates fitted in each of the 10 training data set folds. Symbols indicate regression coefficient; thick and thin lines represent 90% and 95% CI, respectively.

A summary of the accuracy statistics by cross-validation fold as well as the average across folds is given in Table 2. Across all data, the correlation between actual and predicted live weight was 0.76, varying from 0.64 to 0.82 per fold. The linear regression coefficient of actual live weight on predicted live weight across all data was 0.99; per fold, the linear regression coefficient varied from 0.81 to 1.22. The RMSE of prediction of live weight varied from 4.40 to 6.66 kg per fold, with an RMSE across all data of 5.61 kg. Thus, 68% of the live weight predictions were within 5.61 kg of the actual live weight. Bias existed (*P* < 0.05) only in fold 10, where the model underpredicted live weight by 1.77 kg.

**Table 2:** Accuracy statistics (SE in parentheses) reflected by the correlation between predicted weight and actual live weight, the slope coefficient of the regression line, the root mean squared error (RMSE; kg), and mean bias error (MBE; kg) for each of the 10 validation folds as well as when applied across all data^1^

Statistic	Fold										
	1 (n = 61)	2 (n = 61)	3 (n = 61)	4 (n = 61)	5 (n = 61)	6 (n = 61)	7 (n = 61)	8 (n = 61)	9 (n = 61)	10 (n = 53)	All data (n = 602)
Correlation	0.77 (0.08)	0.81 (0.08)	0.72 (0.09)	0.80 (0.08)	0.76 (0.08)	0.80 (0.08)	0.82 (0.07)	0.76 (0.08)	0.64 (0.10)	0.75 (0.09)	0.76 (0.03)
Slope	0.89 (0.10)	1.05 (0.10)	1.06 (0.13)	1.03 (0.10)	0.81 (0.09)	0.99 (0.10)	1.22 (0.11)	1.12 (0.12)	0.81 (0.13)	0.88 (0.11)	0.99 (0.03)
RMSE	4.40	5.10	6.31	4.96	5.00	5.35	6.22	6.66	6.15	5.54	5.61
MBE	−0.98 (0.55)	0.22 (0.66)	−0.94 (0.81)	0.70 (0.63)	−0.61 (0.64)	1.24 (0.67)	0.20 (0.80)	0.34 (0.86)	1.22 (0.78)	−1.77* (0.73)	−0.02 (0.23)

1 n = number of animals in each fold.

* Different (*P* < 0.05) from zero.

Some studies have attempted to predict live weight in growing dairy cattle (Heinrichs et al., 1992; Franco et al., 2017); however, many of these relate to cattle that are months rather than days old (Heinrichs et al., 1992; Franco et al., 2017). Furthermore, much of the available research is based on heifers (Heinrichs et al., 1992; Franco et al., 2017) as opposed to dairy bulls, which were investigated in the present study. Some predictions of live weight in young dairy bull calves from morphological characteristics do, nonetheless, exist (Wilson et al., 1997). The rationale for the greater abundance of studies that predict dairy heifer live weight is to implement regimens, if necessary, to ensure that the heifer achieves her target live weight for a given age, with the end goal of being a target live weight at first calving. The association between live weight at first calving and future performance in dairy cows is well established (Dobos et al., 2004). Because many of these studies predict the live weight of the live heifer, the prediction equations have the luxury of including animal-level features that can be measured directly on the live animal (Heinrichs et al., 1992). Such prediction equations tend to focus on (relatively easily measurable) animal biometric features relating to the morphological characteristics of the heifer, such as heart girth or height (Heinrichs et al., 1992; Franco et al., 2017). The motivation for the present study, however, was to generate a prediction model that could be readily integrated within a decision support tool for planning dairy bull calf management before the initiation of the calving season. Hence, the focus in the present study was on animal-level features that could be available on all animals well in advance of being born. The parity of the dam giving birth will be known; if the sire of the fetus is known, an estimate of genetic merit of the fetus is simply the mean of the parental genetic merit. Although gestation length is not known before birth, predictions based on ultrasound examination of the pregnancy are accurate (Fitzgerald et al., 2015). Irrespective, over- or underestimating gestation length by 5 d equates to an error in predicting age to reach a given weight of just 1 d, although the effect on planning is compounded by the error in the prediction of the actual birth date. Nonetheless, replacing actual realized phenotypic gestation length in the prediction model with EBV for gestation length, or removing gestation length in its entirety from the model, reduced the correlation between actual and predicted live weight by, on average, just 0.001 and 0.004, respectively, across folds. Hence, inaccuracies in the estimation of gestation length will have minimal effect on calf management planning, and, although associated with live weight in the multiple regression prediction model (*P* < 0.001), its marginal contribution to explaining the variability in live weight was small.

With a correlation of 0.76, the accuracy of predicting live weight in the present study could be considered good, albeit generally not as accurate as other live weight prediction models in heifers using live body measures (i.e., correlation >0.96) as reported by Heinrichs et al. (1992). It is, nonetheless, important to remember that the gold standard dependent variable itself is likely to contain error. First, the weighing scales measured only in 1-kg increments. Given that the mean live weight of the calves used in this study was 56.6 kg, this represents almost 2% of the mean. Furthermore, calves will differ in terms of the duration between when they were last fed and when they were weighed or how much they were fed. Calves of the age used in the present study are often fed 15% of their BW in liquid plus roughage (Conneely et al., 2014), which here would equate to, on average, 8.5 kg. These factors therefore contribute to random variability, entering the residual term in the statistical model with consequential effects on prediction accuracy. Despite this variability, 68% of the live weight predictions were within 5.61 kg of the actual live weight of the calves in the present study.

The success of the prediction model developed in the present study is predicated by having genetic evaluations for the traits assessed herein—namely birth size, birth weight, and carcass weight. Not all populations will have such genetic evaluations, although they may have genetic evaluations for similar traits. Although recording birth weight can be time consuming, birth size in Ireland is scored subjectively on a 1-to-5 scale, thus making it amenable for easy and safe recording. Carcass weight information is available on all animals slaughtered in Ireland. Ideally, a genetic evaluation for calf live weight around 4 to 7 wk of age would be available that should correlate well with phenotypic live weight at that age. Weighing all calves at the time of sale could be one approach to achieving the goal of a genetic evaluation for live weight in early life, assuming that other information such as parentage and date of birth is known. Weighing the calves at birth would undoubtedly improve the prediction of live weight some weeks later; however, this is unlikely to happen on most dairy farms, where labor is already in high demand. Nonetheless, when weighing animals at sale, it could also be possible to gather useful ancillary information such as calf quality, calf health, and any other features such as congenital defects.

Phenotypic data do not have to exist for all calves for use in genetic evaluations, just for a sufficient number of calves to achieve high accuracy of selection. The relationship between accuracy of genetic or genomic evaluations and number of records is a function of the heritability of the trait. The reliability of genetic evaluations for an animal with just its own phenotypic record will be the heritability of the trait, which was 0.38 in the present study. There is actually a paucity of heritability estimates in the international scientific literature for live weight in young dairy bull calves. Also important is the potential to estimate herd-specific effects if the herd is treated as either a random effect or a fixed effect in the statistical model. After adjusting for the fixed effects within the model, quite large variability in mean calf live weight existed among herds in the present study as evidenced by the standard deviation in herd effects from the full data set of 4.62 kg. This occurred despite the herds operating in similarly strict spring-calving production systems, implying that the variability could be larger nationally.

The approach in the present study was to estimate live weight for a given age (and other animal-level features). However, Equation 1 can simply be rearranged and, using the estimated model solutions, this can easily be translated to the expected age of a calf to reach a target live weight:

[2] $$\text{age}=\frac{\left[\text{traget weight}-\left(\sum \text{model solutions}\times \text{animal-level features}\right)\right]}{b_{\text{age}}},$$

where age is the expected age for a calf to reach a target live weight (e.g., 50 kg); Σ model solutions × animal-level features is the sum across the animal-specific values for each model feature (except age) times the respective regression coefficients from Equation 1, including the herd random effect; and *b*_age_ is the estimated regression coefficient from Equation 1. Based on Equation 2, when the herd effect was ignored, the average animal (i.e., the mean phenotypic gestation length and EBV of the population for all traits) from a third-parity dam is expected to be 17, 25, and 33 d old when it reaches a target live weight of 50, 55, and 60 kg, respectively. Based on the model applied to the entire data set, the standard deviation in the estimated age to reach 50 kg live weight was 5.2 d. This implies that the mean difference in age to reach 50 kg differed by 18 d between the quickest and slowest 10% of calves. Such a wide range has obvious implications for resource requirements on farm, enabling the fine-tuning of associated management strategies over and above simply assuming that all bull calves could leave the farm at 17 d of age once they weigh 50 kg.

The present study focused on predicting the age when a bull calf will reach a given live weight. Complementary tools based on similar data sources can be used to allocate a value to the calf. Dunne et al. (2021) developed a decision support tool based on selection index theory, which they proposed could be used as a mechanism for assigning a price to an animal, whatever the age, based on a series of relevant traits. Although both the transaction index proposed by Dunne et al. (2021) and the model developed in the present study have similarities, they are highly complementary. Overlaying both systems with an efficient information and communications technology system, including a web service, could revolutionize how dairy bull calves are managed and transacted. This could be particularly useful in scenarios in which it may not be possible to view animals, such as when highly infectious animal diseases are circulating (e.g., foot and mouth) or in the midst of a human pandemic (e.g., COVID-19).

The approach described in the present study could be a useful tool to aid in farm management and could potentially lead to more consistent batches of calves being available for purchase, thereby simplifying the management of these cattle to eventual harvest. The developed model is relatively simple and, if genetic evaluations for some measure of size or live weight exist, can easily be deployed in other jurisdictions once the model solutions are re-estimated. Although the motivation for predicting calf live weight and, by extension, age to reach a given live weight was to aid in resource management, prediction of calf live weight has other uses in calf management, including determining the appropriate feeding level and the appropriate medicinal dosage rates.
